# Unicompartmental knee replacement combined with anterior cruciate ligament reconstruction provides comparable results to total knee replacement with no increased risk of complications

**DOI:** 10.1051/sicotj/2024005

**Published:** 2024-02-28

**Authors:** Claudio Legnani, Enrico Borgo, Vittorio Macchi, Clara Terzaghi, Alberto Ventura

**Affiliations:** 1 IRCCS Istituto Ortopedico Galeazzi, Sports Traumatology and Minimally Invasive Articular Surgery Center Via Monreale 18 20148 Milan Italy; 2 Istituto Clinico Villa Aprica Via Castel Carnasino 10 22100 Como Italy

**Keywords:** Unicompartmental knee replacement, Anterior cruciate ligament, ACL reconstruction, Total knee replacement

## Abstract

*Introduction*: There is controversy about the management of unicompartmental knee osteoarthritis (OA) in young, active patients with anterior cruciate ligament (ACL) insufficiency. This study compares the subjective, radiological, and functional results of total knee replacement (TKR) vs. combined medial unicompartmental knee replacement (UKR) with ACL reconstruction. *Method*: Twelve patients suffering from medial OA and ACL deficiency with varus knee deformity and/or tibial slope <10° and absence of patellofemoral-related problems were eligible for combined UKR and ACL reconstruction (Group A). Twenty-six patients matched for age, male/female ratio and body mass index who received TKR in the same time frame were included as a control group (Group B). Oxford Knee Score (OKS), WOMAC index of osteoarthritis, Knee Osteoarthritis Outcome Score (KOOS), and routine X-rays were used for assessment. *Results*: Ten years after surgery, the mean overall KOOS score, OKS, WOMAC index increased from preoperatively, showing a statistically significant difference (*p* < 0.001). In terms of KOOS, OKS, or WOMAC scores at the most recent follow-up, there was no discernible difference between the groups (*p* = n.s.). Three years following surgery, one female patient in group A received revision TKR due to the lateral compartment’s osteoarthritis developing and the patient’s pain persisting. Concerning radiographic assessment, at the most recent follow-up (average 7.9 years in group A and 8.8 years in group B), there were no radiographic indications of implant loosening or proof of pathologic radiolucent lines. *Conclusions*: UKR combined with ACL restoration offers clinical and radiographic outcomes comparable to TKR 10 years following surgery with no elevated risk of complications.

## Introduction

For the treatment of medial femorotibial osteoarthritis (OA) in conjunction with anterior knee laxity, several surgical treatments are available [1]. Unicompartmental knee replacement (UKR) is generally not recommended in the presence of concomitant anterior cruciate ligament (ACL) deficiency, and traditionally, total knee replacement (TKR) has been safely done in this setting [2]. However, considering these surgical options, UKR has advantages over TKR, including less invasiveness, lower blood loss, bone stock preservation, a quicker recovery, and improved joint kinematics [3–5].

For these reasons, more recently, ACL-deficient patients with isolated medial OA demonstrated to benefit from fixed-bearing UKR implanted with a lower grade of tibial slope in order to compensate for anterior tibial translation [6]. However, to overcome the potential risks of posterior femoral shift brought by ACL insufficiency and increased incidence of aseptic loosening, a surgical strategy allowing concomitant UKR and ACL reconstruction has been successfully proposed (Table 1) [7–19].

**Table 1:** Summary of all available studies on combined UKR and ACL reconstruction.

Author	Year	Patients number	Mean age (range) [SD]	Male/female ratio	Mean follow-up (range) [SD]	Post-op mean outcome score (range) [SD]	Bearing type	Graft type	Complications (rate)
Pandit	2006	15	49.8 (36–60)	13:2	2.8 (2.5–4.3) years	OKS: 46 (37–48)	Mobile	3 BPTB	1 (6.7%) Infection and two-stage revision to a TKR
						KSS objective: 99 (95–100)		11 Hamstrings	
						KSS functional: 96 (85–100)			
						Tegner activity level: 3.8 (3–6)			
Krishnan	2009	9	56 (50–64)	5:4	24 (12–60) months	WOMAC: 24 (21–27)	Fixed	8 BPTB	None
						OKS: 11 (10–12)		1 Hamstrings	
						KSS: 196 (190–200)			
Tinius	2012	27	44 (38–53)	11:16	50 (9–71) months	OKS: 166 [12.1]	Fixed	Hamstrings	None
						KSS objective: 83.2 [6.8]			
						KSS functional: 82.7 [8.2]			
Weston-Simons	2012	51	51 (36–67)	40:11	60 (12–120) months	OKS: 41 (17–48)	Mobile	(?) BPTB	1 (2%) Infection and two-stage revision to a TKR
						AKS functional score: 95 (45–100)		(?) Hamstrings	1 (2%) Bearing dislocation
						AKS Objective Score: 75 (25–95)			1 (2%) Symptomatic lateral osteoarthritis and conversion to TKR
						Tegner activity level: 3.5 (1–5)			
Tian	2016	28	50.5 (41–60)	10:18	52 (24–96) months	OKS: 43 [4.2]	Mobile	Hamstrings	2 (7%) Bearing dislocation
						KSS Objective: 84.5 [6.3]			
						KSS Functional: 86.9 [5.3]			
						Tegner activity level: 5.3 [0.8]			
Iriberri	2018	8	52 (42–60)	5:3	175 (117–258) months	KSS: 154 (102–200)	Fixed	Hamstrings	1 (12.5%) Symptomatic lateral osteoarthritis and conversion to TKR
						WOMAC: 26 (1–52)			1 (12.5%) external meniscus tear repair
						VAS: 3 (0–7)			
Tecame	2019	24	47.8 (41–53)	20:4	53 [8.3] months	WOMAC: 79.3 [7.3] mobile, 81.3 [7.6] fixed	9 Mobile	Hamstrings	None
			48.4 (43–54)		42 [6.7] months	KSS functional: 86.2 [6.2] mobile, 84.7 [5.9] fixed	15 Fixed		
						KSS objective: 73.4 [9.3] mobile, 77.3 [10.5] fixed			
Kennedy	2019	75	52.6 (36–71)	59:16	6.4 (1–15) years	OKS: 41 (11–48)	Mobile	Hamstrings	3 (3.9%) revisions to TKR
						Tegner activity level: 3.6 (0–8)			
Ventura	2019	12	54 [3.9]	8:4	7.8 (6–10) years	KOOS: 80.2 [11.7]	Fixed	Hamstrings	1 (8.3%) Symptomatic lateral osteoarthritis and conversion to TKR
						OKS: 42.4 [8.9]			
						WOMAC: 84.9 [9.3]			
						AKS objective score: 75 [13.5]			
						AKS functional score: 88 [16.2]			
Kurien	2022	24	48.8 (38.3–71.0)	16:8	5.1 (1.3–12.8) years	Lysholm: 92 (90–96)	Fixed	(?) BPTB	1 (4.1%) undisplaced anterior cortex fracture of the medial tibial plateau and 1 (4.1%) iatrogenic Grade 2 injury to the medial collateral ligament treated conservatively
						OKS: 46 (44–47)		(?) Hamstrings	
						Tegner activity level: 3.96 [0.98]		(?) Allograft	
						VAS: 0 (0–0)			
Jaber	2023	23	48 (44–69)	18:5	10 (6–14.5) years	Lysholm: 85.5 (44–100)	Mobile	Hamstrings	2 (8.6%) revisions with conversion to total knee arthroplasty at 6 and 12 years postoperatively
						OKS: 40 (29–48)			
						Tegner activity level: 3.6 (0–7)			
						VAS: 1.3 (0–5)			
						UCLA Activity Level: 6.7 (4–8)			
						AKS objective score: 91.5 (74–100)			
						AKS functional score: 90 (50–100)			
Foissey	2023	10	57 (48–70)	2:8	45 (24–66) months	IKS knee score: 96 (88–100)	Fixed	Hamstrings	2 (20%) re-operations due to postoperative stiffness
						IKS function score: 93 (74–100)			
						Tegner activity level: 4.5 (3–6)			

UKR: unicompartmental knee replacement; ACL: anterior cruciate ligament; TKR: total knee replacement; SD: Standard deviation; BPTB: Bone-Patellar tendon-Bone; KOOS: Knee Osteoarthritis Outcome Score; OKS: Oxford Knee Score; KSS: Knee Society Score; WOMAC: Western Ontario and McMaster; AKS: American Knee Society; VAS: Visual Analog Scale; UCLA: University of California at Los Angeles; IKS: International Knee Society.

Satisfactory mid-term results of this surgical procedure have been reported, although this is a technically demanding procedure which may carry drawbacks such as impingement with the neoligament and improper sizing of the prosthetic tibia, and the relatively high rate of complications reported in some studies may limit this surgical option to become mainstream [19, 20].

This study compares the subjective, radiological, and functional results of combined medial UKR with ACL reconstruction vs. TKR. The hypothesis was that the combined treatment may offer comparable or superior outcomes without adding postoperative complications.

## Patients and methods

### Study design

Fourteen consecutive cases suffering from medial OA and ACL deficiency were treated from 2006 to 2010 with combined UKR and ACL reconstruction. Twelve of them (86%) were successfully monitored up to 10 years after surgery. Prospectively collected clinical outcomes were retrieved from the senior’s author database and retrospectively analyzed. Patients were matched 1:2 to a control group of 24 patients who received TKR for primary idiopathic OA over a period of 2 years (2009–2011) having the same average follow-up (10.0 years). Regarding age, the male-to-female ratio, and body mass index (BMI), the two groups were comparable. BMI > 30, OA involving other compartment than medial, varus knee deformity and/or tibial slope >10°, presence of patellofemoral-related problems were exclusion criteria for UKR. Every time OA was expanded to many compartments, TKR was carried out.

In group A, the mean age at surgery was 54 years (SD: 3.9), while in group B, it was 55.8 years (SD: 4.2) (Table 2). A single surgeon carried out each procedure. Written informed consent was obtained. The study was carried out in accordance with the recommendations made by the Institution’s Ethical Committee and conducted according to the Strengthening the Reporting of Observational Studies in Epidemiology (STROBE) guidelines. Data regarding clinical and radiological assessment up to 10 years after surgery were retrieved from the senior’s author database. The most recent follow-up was considered. Ethical approval was not required for the follow-up of this observational analytic study with a retrospective design on well-established surgical procedures, as the patients’ reported outcomes used are part of routine follow-up at the authors’ institution.

**Table 2:** Patient demographics and anthropometric data.

	UKR + ACL reconstruction	TKR
No. of patients	12	24
Gender		
Male	8	16
Female	4	8
Mean age at surgery (SD) (year)	54.0 (3.9)	55.8 (4.2)
Mean BMI (SD)	25.9 (2.4)	26.2 (2.2)

UKR: unicompartmental knee replacement; ACL: anterior cruciate ligament; TKR: total knee replacement; SD: standard deviation; BMI: Body Mass Index.

### Surgical procedure and rehabilitation protocol

In the case of UKR combined with ACL reconstruction, surgery was performed as previously described [9] using Allegretto unicondylar fixed-bearing prosthesis (Zimmer-Biomet Orthopedics, Warsaw, IN, USA) and autologous hamstring tendon grafts fixed proximally with either a RIGID-FIX equipment (DePuy Mitek, Raynham, MA, USA) or a Retrobutton device (Arthrex, Naples, FL). The distal fixation was achieved through a BioRCI screw (Bioabsorbable Rounded Cannulated Interference, Smith & Nephew Inc. Andover, MA, USA).

All patients in group B underwent medial parapatellar arthrotomy for implantation of a cemented posterior stabilized Vanguard Complete Knee System (Zimmer-Biomet Orthopedics) without patellar resurfacing. The day following surgery, a brace-free post-operative rehabilitation plan was initiated. It included joint motion exercises, an immediate return to full knee extension, and progressive weight-bearing ambulation with crutches. Walking with some weight bearing was permitted for the first four weeks while using two crutches.

### Follow-up assessment

The assessment included the Knee Osteoarthritis Outcome score (KOOS), Oxford Knee score (OKS), and Western Ontario and McMaster (WOMAC) index of OA. A radiological assessment was conducted with standard radiographs in order to get information about any presence of loosening of the components. Radiolucencies were judged as physiological or pathological according to criteria expressed by Goodfellow et al. [21].

### Statistical analysis

SPSS Statistics for Windows^®^, Version 21.0 (IBM Corp., Armonk, NY) was used to analyze the data that had been extracted. The pre-operative and follow-up status, as well as any differences between the two groups, were compared using the Wilcoxon signed-rank test. Differences were deemed statistically significant with *p*-value < 0.05.

## Results

Overall, no major complications (fractures, infections, thromboembolism) were reported.

Superficial wound dehiscence occurred in two patients in group B, while stiffness was reported in three patients following TKR. One female patient in group A got a revision TKR with symptom relief three years after surgery because of pain persistence and OA development in the lateral compartment.

Post-operatively, there was a statistically significant change, as evidenced by increases in the mean overall KOOS score, OKS, and WOMAC index (*p* < 0.001, Table 3).

**Table 3:** Comparison between pre-operative and follow-up status.

	UKR + ACL reconstruction			TKR		
	Pre-operative	Follow-up	*p*-value	Pre-operative	Follow-up	*p*-value
KOOS (mean, SD)	62.4 (8.1)	80.1 (9.8)	<0.001	59.8 (9.2)	79.4 (6.0)	<0.001
OKS (mean, SD)	28.8 (10.1)	42.6 (9.0)	<0.001	27.4 (8.6)	40.1 (6.6)	<0.001
WOMAC score (mean, SD)	71.9 (11.5)	84.6 (8.8)	<0.001	68.9 (5.2)	83.0 (3.5)	<0.001

SD: standard deviation; UKR: Unicompartmental Knee Replacement; ACL: anterior cruciate ligament; TKR: total knee replacement; KOOS: Knee Osteoarthritis Outcome Score; OKS: Oxford Knee Score; WOMAC: Western Ontario and McMaster University Osteoarthritis Index.

Group B scored lower on the baseline questionnaires, although the difference was not statistically significant. At the latest follow-up, there was no significant difference between the groups concerning KOOS, OKS, or WOMAC scores (*p* = n.s., Table 4).

**Table 4:** Comparison between groups at final follow-up.

	UKR + ACL reconstruction	TKR	*p*-value
KOOS (mean, SD)	80.1 (9.8)	79.4 (6.0)	0.78
OKS (mean, SD)	42.6 (9.0)	40.1 (6.6)	0.35
WOMAC score (mean, SD)	84.6 (8.8)	83.0 (3.5)	0.44

SD: standard deviation; UKR: Unicompartmental Knee Replacement; ACL: anterior cruciate ligament; TKR: total knee replacement; KOOS: Knee Osteoarthritis Outcome Score; OKS: Oxford Knee Score; WOMAC: Western Ontario and McMaster University Osteoarthritis Index.

Radiographic follow-up was performed up to 10 years after surgery, although for some patients most updated X-rays were taken at 5 years post-operatively. Thus, for group A, the average radiographic follow-up was 7.9 years (SD: 2.6), while for group B it reached 8.8 years (SD: 2.2). At the most recent follow-up, there were no radiographic indications of implant loosening or pathologic radiolucent lines.

## Discussion

The most significant conclusion of the current study is that UKR combined with ACL reconstruction offers clinical and radiographic results comparable to TKR 10 years following surgery with no increased risk of complications. In the medium-long term, both therapies enhance postoperative clinical and functional results. At the follow-up, there were no statistically significant differences between the two groups in terms of functional results.

Foissey et al. reported on 10 patients after robotic-assisted UKR combined to hamstring ACL reconstruction after an average follow-up of 45 months. The mean post-operative International Knee postoperative function score was 93, mean Tegner score was 4.5 [10]. Two patients underwent arthroscopic arthrolysis following postoperative arthrofibrosis. In the study by Tian et al. on 28 patients treated by combined UKR and ACL reconstruction, 4 years after surgery, the average OKS was 43, and Tegner activity level reached the mean value of 5.3 [7].

Jaber et al. followed up for 10 years 23 patients who had undergone mobile-bearing UKR combined with ACL reconstruction [12]. The average Lysholm score was 85.5, while the mean OKS was 40. None of the patients reported knee instability and all of them returned to sporting and physical activities. The authors reported a survival rate of 91.4% at 14.5 years. Similarly to our case series, one patient received a conversion procedure one year after surgery due to the progression of symptomatic lateral knee OA.

In the study by Kurien et al., 24 patients underwent simultaneous ACL reconstruction and medial UKR. After an average follow-up of 5.1 years, the mean OKS score was 46, while the average Lysholm score was 92. Consistently to our study, fixed-bearing UKR was chosen to avoid the risks of bearing dislocation [11].

According to our results, 10 years after surgery, there was no discernible difference between the two groups regarding KOOS, OKS or WOMAC scores, showing that combined UKR and ACL reconstruction allow comparable outcomes to TKR. This suggests that this combined procedure is effective when approaching relatively young and active individuals with ACL insufficiency and advanced unicompartmental knee OA. Additionally, the advantages of this combined approach over TKR are less invasiveness and blood loss, and better knee kinematics.

Nowadays, patients suffering from knee OA are more likely to be willing to return to physical activity and active daily living [22]. Advancements in surgical methods and prosthetic designs may enable patients to return to pre-operative levels of physical activity [23].

Drawbacks of this combined procedure include the fact that it is technically challenging, and carries potential risks of post-operative arthrofibrosis, ACL graft impingement, and tibial base plate under-sizing. To reduce these disadvantages, simultaneous ACL reconstruction and UKR should be preferable compared to a staged one. In fact, as suggested by Pandit et al., a more vertical drilling and lateral exit of the tibial tunnel aperture can prevent graft impingement of the neo-ligament [13]. Another potential issue related to this surgical approach is the longer post-operative rehabilitation period compared to TKR. To overcome this drawback, compliance from the patient is essential for a successful outcome. According to Derreveaux et al., in physically active patients with unicompartmental knee OA, combining UKR with additional surgery to manage relative contraindications to TKR is an effective strategy that yields satisfactory results at short-term follow-up. According to the authors, it should only be used on patients who have received comprehensive counseling about all available therapeutic alternatives and for whom TKR is likely to yield disappointing outcomes [24].

Eventually, concerning patients’ selection, only patients with OA secondary to ACL deficiency should be eligible for this combined procedure. In fact, for patients whose main problem is cartilage deterioration and secondary ACL instability, TKR should be preferred due to the presence of contracture to the medial collateral ligament and wear on the other compartments [12, 20].

The present study has some limitations, including its retrospective character and the limited study population, due to the highly selective patient selection criteria required for this operation. The small sample size may not have allowed for the detection of small differences between groups. Postoperative results showed a tendency in favour of ACL reconstruction combined with UKR, although not statistically significant. Further multi-center studies with larger cohorts are warranted to find more reliable results and conclusions.

## Conclusions

UKR combined with ACL reconstruction is a valid therapeutic option for young and active patients with a primary ACL injury who develop secondary OA and confirms subjective and objective clinical improvement up to 10 years after surgery.

## Data Availability

Data are available on request from the authors.
